# Experimental Comparison of the High-Speed Imaging Performance of an EM-CCD and sCMOS Camera in a Dynamic Live-Cell Imaging Test Case

**DOI:** 10.1371/journal.pone.0084614

**Published:** 2014-01-03

**Authors:** Hope T. Beier, Bennett L. Ibey

**Affiliations:** 1th Human Performance Wing, Bioeffects Division, Air Force Research Laboratory, Fort Sam Houston, Texas, United States of America; Arizona State University, United States of America

## Abstract

The study of living cells may require advanced imaging techniques to track weak and rapidly changing signals. Fundamental to this need is the recent advancement in camera technology. Two camera types, specifically sCMOS and EM-CCD, promise both high signal-to-noise and high speed (>100 fps), leaving researchers with a critical decision when determining the best technology for their application. In this article, we compare two cameras using a live-cell imaging test case in which small changes in cellular fluorescence must be rapidly detected with high spatial resolution. The EM-CCD maintained an advantage of being able to acquire discernible images with a lower number of photons due to its EM-enhancement. However, if high-resolution images at speeds approaching or exceeding 1000 fps are desired, the flexibility of the full-frame imaging capabilities of sCMOS is superior.

## Introduction

In live cell microscopy, the ability to dynamically image weak and rapidly changing fluorescence signals is often desired. The most critical system component for this type of imaging is the fluorescence camera, which often leaves scientists to decide between one of two leading technologies. Electron Multiplying Charge Coupled Device (EM-CCD) cameras use an on-chip gain process to multiply the number of electrons prior to digitization thus requiring a very low numbers of photons to produce an image [1]. New scientific-grade CMOS (sCMOS) cameras report read noise levels of 1–2 e^−^, which despite being around an order of magnitude higher than EM-CCDs, may achieve similar low-light sensitivity as they do not suffer from electron multiplication noise [2]. Efforts have been made to directly compare EM-CCD and sCMOS camera technologies for biological imaging applications [2]–[7]. Many of these studies have focused on the theoretical aspects of camera function and not real-world biological imaging applications [6], [7]. Other studies have compared the technologies using real-world examples, but mostly focus on low-light sensitivity and localization capability of the cameras with little concern for acquiring images at the highest possible frame-rate [2]–[5].

In this paper, we examine two key imaging parameters, SNR and image acquisition rate, to characterize the performance of high-frame-rate EM-CCD and sCMOS camera technologies for high-speed fluorescence image acquisition. We examine the trade-off between temporal and spatial information using a dynamic situation in which the fluorescence in living cells is rapidly changing (∼1 ms). To elicit a rapid fluorescence change, we apply an intense nanosecond-duration electrical stimulus to living cells, which has been shown to rapidly increase the intracellular calcium concentration [8], [9]. We use this model system to compare the response at similar acquisition rates and spatial resolution for the two test cameras. The goal of this case-study is to provide researchers with useful information to allow them to make a more educated assessment as to which camera technology will best meet their high-speed imaging needs. For this study, we will compare the current version of each camera technology with the highest frame-rate.

## Results

### High-speed Images of Calcium Influx

As a test case for imaging a dynamic fluorescence event in living cells, we loaded rodent neuroblastoma cells with Calcium Green-1 AM ester (CaGr). A pulsed electric field was delivered to individual cells by a microelectrode [10]–[13]. This stimulus is known to cause a rapid increase in intracellular calcium concentration [8], [9]. The cells were illuminated by the 488 nm line of an Argon-Krypton ion laser with the beam expanded prior to the microscope to allow for illumination of the entire cell. Images were acquired through a 100X oil-immersion objective on an inverted microscope with the EM-CCD or sCMOS camera attached to the epi-fluorescence camera port [14].

**Figure 1:**
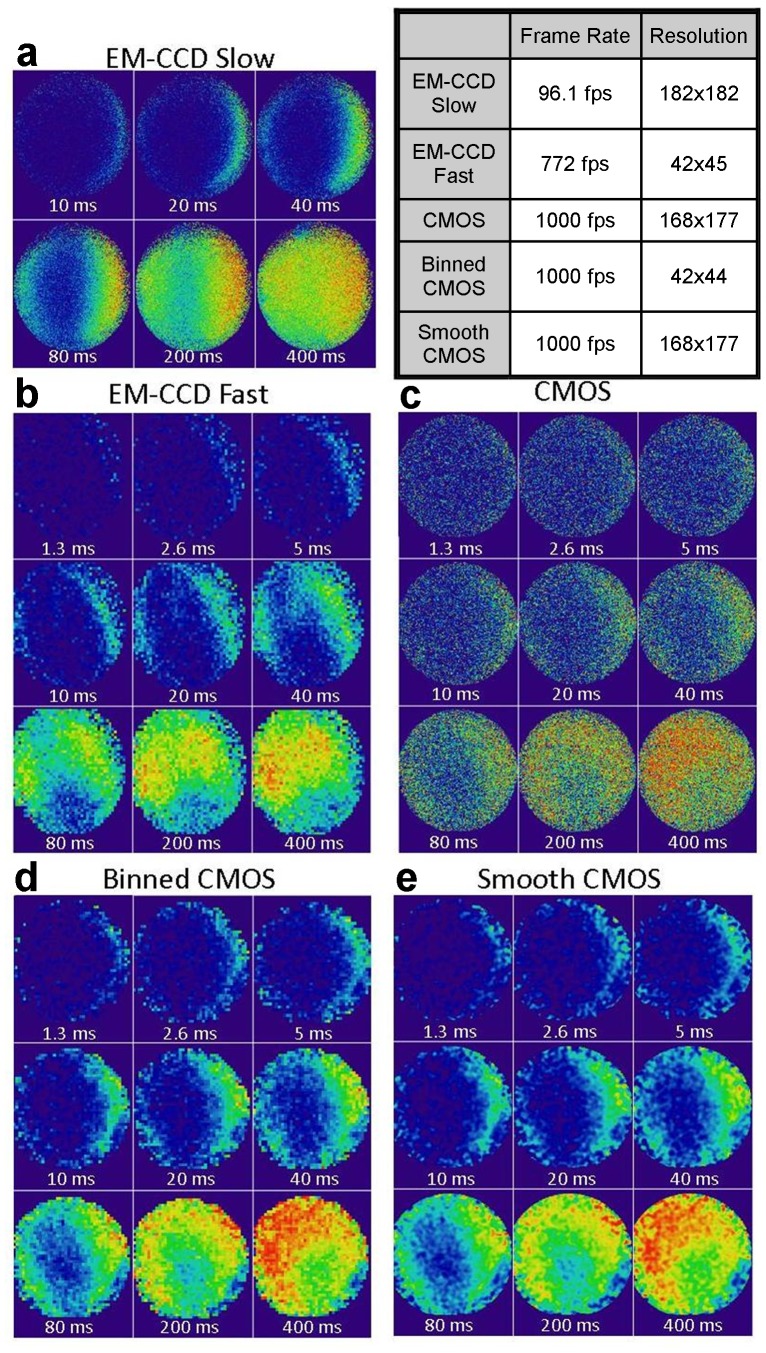
Temporal snapshots of the calcium influx into NG108 cells. Images are taken with each camera with different acquisition parameters.

The high-speed calcium kinetics for the NG108 cells exposed to the electrical stimulus for each camera are shown in Figure 1. The NG108 cells selected for these exposures where round in morphology and the images have been cropped to the edges of the cells so that the initial influx of calcium is more apparent. In these images, the background intensity is subtracted and then the increase in fluorescence (ΔF/F) is determined for each pixel in each temporal image. The average of the ten frames recorded prior to the electrical stimulus is used as the baseline, F. To increase the acquisition rate, the EM-CCD possesses a cropped sensor mode, which shrinks the image to a user-defined region-of-interest (ROI). An external mask darkens the image outside of this region of interest so that no photons fall on the unused pixels. This approach, along with pixel binning, allows the image acquisition rates to be increased from 56 frames per second at full frame to >1000 fps for binned ROIs. In image series 1a, the cellular response as imaged by the EM-CCD camera using the crop mode with an area of 182×182 pixels, but no binning is shown. For these acquisition parameters, we obtained an image acquisition rate of 96.1 fps. These image parameters represent the maximum speed obtainable with maximum resolution as the priority (no pixel binning) given our microscope set-up. It is clear that for the first image after the pulse, at 10 ms, that fluorescence has already increased along the right side of the cell. This influx of calcium continues over time to fill the cell as shown by the subsequent images. By using the 4×4 binning option of the camera, we are able to increase the image acquisition rate to 772 fps, at a loss of image resolution, as shown in the image series in 1b. Using a different NG108 cell with a similar response as the previous, we can see that the initial influx in calcium actually occurs as quickly as 1.3 ms after the pulse, information lost with the slower acquisition.

Calcium influx into equivalent NG108 cells exposed to the same electrical stimulus was then imaged by the sCMOS camera. Image series 1c shows the temporal change in the fluorescence intensity of CaGr for the raw sCMOS image. While the increase in calcium is evident, it is more difficult to resolve by eye due to the salt-and-pepper nature of the sCMOS image. This appearance is a result of the way the pixels are read out in a sCMOS camera. Each pixel in our tested sCMOS camera has separate readout amplifier. Each column is then passed through a separate amplifier and analog-to-digital converter. Thus, the camera has apparent increased pixel-to-pixel variability, as compared to the EM-CCD images. However, despite the noise in the image, the sCMOS allows acquisition of the higher resolution images at 1000 fps, whereas the EM-CCD was limited to 96 fps.

To better equate the imaging capabilities of the two cameras, two post-processing applications were applied to the sCMOS images. First, in image series 1d, 4-by-4 pixel binning was applied to the raw sCMOS image. This process reduces the pixel resolution to equivalent resolution of the highest acquisition rate EM-CCD images. As a result of this processing, the change in fluorescence is much clearer. As a second processing approach, in series 1e, the pixels were smoothed with a Gaussian blur filter with a radius of 2 in ImageJ [15]. This smoothing process maintains the number of pixels in the image but reduces the spatial information as each pixel-intensity is impacted by those around it.

### Comparison of Theoretical Values

The image quality of a camera can be described by the SNR versus the number of input photons [2]. For an input photon number, N*_sig_*, the SNR may be calculated as:
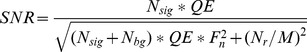
(1)


In this equation, QE is the quantum efficiency of the camera, which is the probability that a photon incident on the pixel will be converted to a photoelectron. N*_bg_* is the signal from background photons and includes background fluorescence and residual excitation photons. At low light levels, the primary noise source is the readout noise, N*_r_*. EM-CCD cameras use an on-chip gain process to multiply the number of electrons before digitization, indicated by the EM gain factor M, which increases the signal by orders of magnitude above the read noise, effectively eliminating this noise source. However, the electron multiplication introduces an additional noise source, F*_n_*, that has been calculated to be 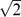 [1]. Since the sCMOS does not have EM gain, the value of F*_n_* is 1.

**Figure 2:**
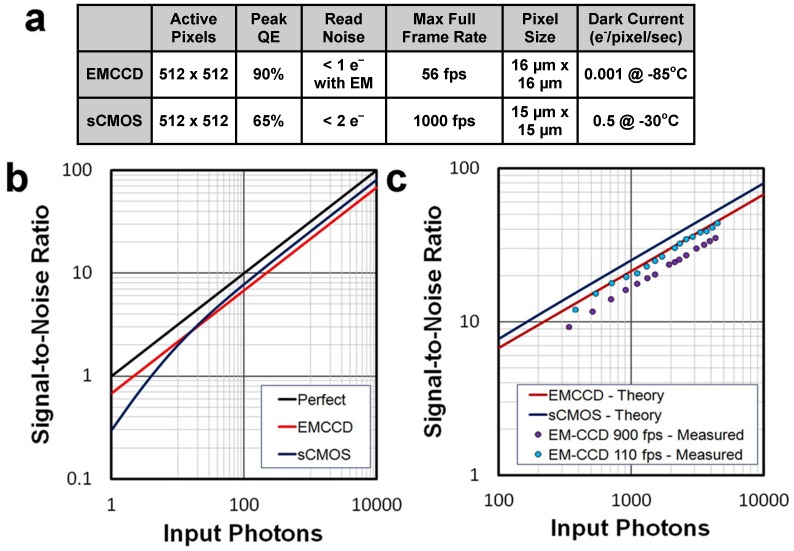
Comparison of the Theoretical versus the Measured SNR. a) Manufacturer provided parameters for the two cameras. A 1024×1024 version of the sCMOS is offered; however, the 512×512 version was tested. b) Theoretical SNR for the two cameras given their manufactured specified parameters as compared to a perfect camera. c) Theoretical comparison of the two cameras compared with the measured SNR of the EM-CCD and two average acquisition rates.

As a first comparison, we compared the theoretical SNR as a function of input photon numbers for the two cameras, given the manufacture’s specifications, which are provided in the table in 2a. For this comparison, we assumed that the background noise is negligible. We also did not account for any dark noise, which results from thermally-generated electrons, as our acquisition rates were so short that it should not be a significant noise contribution. In Figure 2b, these SNR curves are plotted against the SNR for a perfect camera, which is assumed to have a QE of 1 and no multiplication noise or readout noise. At extremely low light levels, the EM-CCD theoretically out performs the sCMOS, until at a point around 15 photons/pixel in which the sCMOS provides slightly better SNR.

We then used the post-processing function of the EM-CCD to convert the image counts to number of photons and compared these values to the theory. To calculate the SNR, the background number of photons was first subtracted; this subtraction was mirrored in the theoretical calculation by assuming the background signal is zero. The values for each pixel for a 150 ms period after the electrical stimulus was then fit to a smooth polynomial and the residuals were considered to be the noise. The SNR was determined and then binned and averaged for six lower acquisition rate and six higher acquisition rate images. As seen in Figure 2c, for the slower acquisition rate images (average 110 fps) the experimental SNR falls closely along the predicted SNR value. For higher acquisition rates (average 900 fps), the SNR falls off slightly.

### Signal-to-Noise in Terms of Fluorescence Change

**Figure 3:**
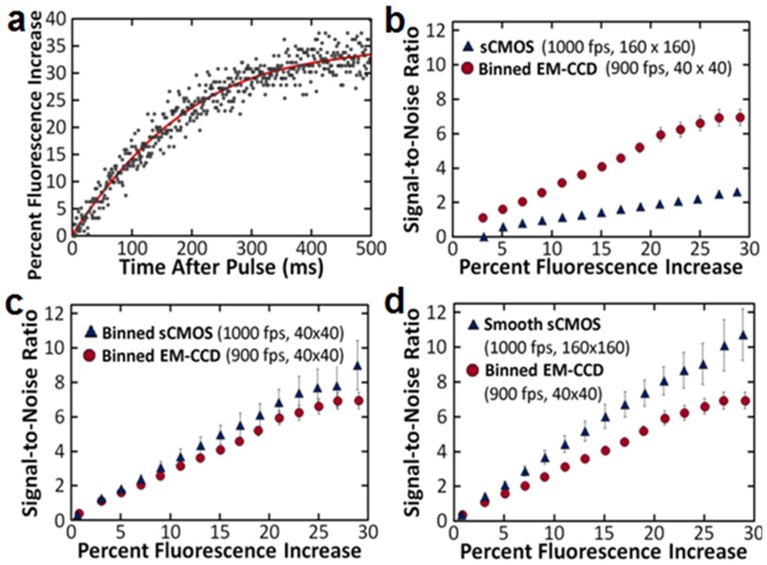
Signal-to-noise comparison between the EM-CCD and CMOS cameras. a) A representative plot showing the measured fluorescence increase at each time-point and polynomial fit for an individual pixel. b) SNR comparison of the raw EM-CCD and sCMOS images with similar acquisition rates. c) SNR comparison with the sCMOS images binned to achieve similar resolution as the EM-CCD. d) SNR comparison with Gaussian smoothing applied to the sCMOS image.

To determine how these cameras will function in a dynamic live-cell imaging situation in which the fluorescence signals are changing as quickly as within 1 ms, we compared the signal-to-noise of a rapidly changing fluorescence signal in terms of the percent change in fluorescence. An approximately 10% slice in the center of each cell was selected and the percent change in fluorescence is found for each pixel at each time-point. Figure 3a shows a typical trace from one of the pixels. The average of the ten frames prior to the electrical stimulus was set as the baseline intensity for each pixel. As the fluorescence signal results from a rapid influx of calcium from the extracellular solution (2 mM Ca^2+^) into the intracellular region of the cell (∼100 nM Ca^2+^ initially), the fluorescence is expected to steadily increase. Thus, this increase in fluorescence is assumed to be a smooth function and is fit to a polynomial function by minimizing the sum of the squares of the residuals as shown by the red line in Figure 3a. The residuals are then determined for each time-point as the noise. The average signal-to-noise for discrete fluorescence increase bins is calculated and further averaged for images of six NG108 cells for each acquisition variable. These results are compared in Figure 3.

First, in Figure 3b, the signal-to-noise for the high-acquisition rate EM-CCD images are compared for the raw sCMOS images. For these images, the acquisition rates are similar (1000 fps for the sCMOS vs. an average of 900 fps for the EM-CCD); however the sCMOS has approximately four times the resolution of the EM-CCD. While the signal-to-noise of the EM-CCD is significantly higher than the sCMOS, the pixels for the EM-CCD have been effectively averaged through the 4×4 binning required to increase the acquisition rate. Thus, for a fairer comparison, the sCMOS image is binned so that the resolutions are equivalent. In this case, as shown in Figure 3c, the signal-to-noise ratio is effectively identical for the two imaging approaches. Because the sCMOS allowed acquisitions at higher resolutions, some spatial information can be retained and the signal-to-noise improved for the sCMOS by applying a Gaussian blur smoothing function to the raw sCMOS image as shown in Figure 3d. This processing improves the signal-to-noise of the image to better than the EM-CCD at higher percent fluorescence values while maintaining near equivalent sensitivity at the lower changes.

## Discussion

The key factor in image quality is the SNR, which is effectively the signal divided by the standard deviation of the total noise at each pixel. The SNR determines how well the features of an image can be identified and allows for a quantitative means to evaluate the capability of a camera. The EM-CCD performed exceptionally close to the manufacture’s specifications in terms of signal-to-noise as a function of input photons for the slower (100 fps) acquisition rates. The slight decrease in signal-to-noise seen with our experimental data, as compared to theory, was likely due to a combination of shot-noise in our excitation laser and natural fluctuations in the actual fluorescence signal stemming from our living samples. Conversion parameters to translate number of counts to photons where not provided for the sCMOS. However, by comparing the SNR response of the two cameras directly as in Figure 3b, it appears that we were unable to achieve the theoretical SNR with the uncooled demonstration version of the sCMOS camera that matched the manufactures specifications. This result may be due to increased dark noise from the camera not being cooled. Additionally, while EM-CCD read noise is typically Gaussian, sCMOS cameras typically possess a skewed distribution. While the reported read noise may be fairly low, the camera may have a large number of pixels on the tail of this distribution with larger noises that appear as random brighter and darker pixels in the images, which was evident by the speckle pattern of the images. The large number of noisy pixels may be driving up the experimentally-derived signal-to-noise ratio. We should note that other sCMOS sensor manufactures base their sensors on smaller pixel formats (3.63 *µ*m by 3.63 *µ*m to 6.5 *µ*m by 6.5 *µ*m) with distinct noise properties, and thus may be less susceptible to the large speckle pattern noise that limits these images. However, the maximum full frame rates for these sensors are lower (30 to 100 fps).

In some instances, image acquisition is limited by the available photons from the sample. While the images demonstrated here displayed a sufficient number of photons to acquire high-speed images of fluorescence dynamics, the irradiance of our laser source was increased by a factor of four from our typical settings used for the EM-CCD to acquire discernible images on top of the salt-and-pepper noise of the sCMOS camera. The EM-CCD images used in this study were also acquired at this increase irradiance. Thus, if images with low number of photons (single to a few hundred photons/pixel/image) are desired, the EM-CCD may be advantageous. Full resolution images can be acquired at rates approaching 100 fps with the ability to detect very low signal counts through EM multiplication. This advantage is maintained for even brightly-fluorescent indicators as greater signal-to-noise allows the use lower dye concentrations and lower excitation intensities to reduce phototoxicity and photobleaching.

If higher acquisition rates are desired and sufficient signal will be available, the sCMOS prevails with its flexibility. Full-frame images can be acquired at 1000 fps. While the raw sCMOS images demonstrated relatively poor SNR, the higher resolution acquisition at the higher frame rate left open the possibility to improve the SNR with post-processing. In essence, while it is possible to compensate for SNR the sCMOS is lacking through post-processing, should more fidelity be required, it is more difficult to acquire the spatial resolution that the EM-CCD is lacking at high acquisition rates. Thus, in conclusion, for extremely dim samples the EM-CCD is desired due to its ability to acquire lower-intensity signals; however, higher resolution images approaching 1 ms/frame are not possible. For high-resolution images at rates closer to 1000 fps, the flexibility of the sCMOS is preferred.

## Materials and Methods

### Imaging System

Excitation of intracellular fluorescent dye is accomplished with the 488 nm line from an Argon-Krypton ion laser (Coherent). To allow for adjustable illumination diameters of 40–150 *µ*m at the sample plane, the beam is routed through a variable beam expander The laser beam is then coupled through the epi-illumination pathway of an inverted microscope (Olympus X51) and through a 100X (1.4 NA, oil) objective. Fluorescence emission is collected by the same objective and after filtering (Semrock), delivered to the EM-CCD (Andor iXon3 897) or sCMOS (Photonis xsCell) camera. The irradiance at the cell plane was limited to 10 W/cm^2^ and cell exposure was limited to the acquisition period by an electronic beam shutter. This precaution was introduced to limit photobleaching and phototoxicity within the sample. Timing of the imaging system, laser irradiation, and electric-pulse delivery was controlled with a digital delay generator (Stanford Research Systems), which enabled precise timing of the experiment with jitters measured to be only 4 ns for the pulser and 1 *µ*s for the EM-CCD camera. The demonstration model sCMOS camera was not yet equipped with a trigger function so imaging acquisition so timing was based on the opening of the laser shutter, with a jitter of ∼1.5 ms. The addition of an adjustable region-of-interest cropping and binning allow us to acquire images at a speeds up to 1 ms/frame with the EM-CCD. An optical mask is used to black-out the pixels except for those in the bottom left of the sensor area. Two-by-two and four-by-four pixel binning is then further used to increase the acquisition rate.

### Live-Cell Test Case

To image calcium release into cells with high-speed, rodent neuroblastoma cells (NG108-15, ATCC) were cultured according to ATCC protocol and allowed to adhere to a glass bottomed poly-L-lysine coated 35 mm culture dish for 24 hours. A standard loading buffer solution consisting of 2 mM MgCl_2_, 5 mM KCL, 10 mM HEPES, 10 mM Glucose, 2 mM CaCl_2_, and 135 mM NaCl was created with a pH of 7.4 and osmolarity of 290–310 mOsm. To load the cells, Calcium Green 1 AM ester (CaGr, Invitrogen) was added at ∼3 *µ*M in loading buffer and incubated at room temperature for 30 minutes. After 30 minutes of loading, the loading buffer was removed and replaced with fresh buffer. Pulse electric fields with 600-ns pulse width and applied voltage of 500 V were delivered to individual cells by a micro-electrode that consisted of two parallel 150-*µ*m diameter tungsten wires with a gap spacing of ∼100 *µ*m.

### Image Processing and Statistical Analysis

Gaussian blur smoothing with a radius of 2 pixels was applied to the indicated sCMOS images in ImageJ [15]. All other image processing and analysis was accomplished in MATLAB*^TM^*. The average pixel counts for images acquired with the excitation laser shutter were determined as the background. The background was then subtracted from all images. The baseline fluorescence, F, was determined for each pixel by averaging the ten frames recorded prior to the electrical stimulus. Fluorescence response was determined as a percent increase at each pixel, ΔF/F, for each time point. The fluorescence signal in terms of both number of pixels and percent change was considered to be a smooth function and fit to a forth-order polynomial by minimizing the sum of the squares of the residuals in MATLAB*^TM^* using the built-in minimum search function. The resulting function was indicated as the signal with the residuals as the noise. Signal-to-noise was calculated as the signal divided the square root of the sum of the squared residuals.
